# Increased CD8^+^ T cell responses to apoptotic T cell-associated antigens in multiple sclerosis

**DOI:** 10.1186/1742-2094-10-94

**Published:** 2013-07-27

**Authors:** Francesco Lolli, Helene Martini, Alessandra Citro, Debora Franceschini, Emilio Portaccio, Maria Pia Amato, Rosella Mechelli, Viviana Annibali, John Sidney, Alessandro Sette, Marco Salvetti, Vincenzo Barnaba

**Affiliations:** 1 Dipartimento di Scienze Biomediche, Sperimentali e Cliniche and Neurofarba, Università of Firenze, Azienda Ospedaliera di Careggi, Largo Brambilla 6, 50134, Firenze, Italy; 2 Dipartimento di Medicina Interna e Specialità Mediche, Sapienza Università di Roma, Viale del Policlinico 155, 00161, Rome, Italy; 3 Centre for Experimental Neurological Therapies, Department of Neuroscience, Mental Health and Sensory Organs (NESMOS), Sant’Andrea Hospital, Sapienza Università di Roma, Via di Grottarossa 1039, 00189, Rome, Italy; 4 La Jolla Institute for Allergy and Immunology, 92121 San Diego, California, USA; 5 Istituto Pasteur, Fondazione Cenci Bolognetti, 00185 Rome, Italy; 6 Laboratorio di Virologia Molecolare, Fondazione Andrea Cesalpino, 00187, Rome, Italy

**Keywords:** Apoptosis, CD8^+^ T cells, Multiple sclerosis

## Abstract

**Background:**

Here, we evaluated the hypothesis that CD8^+^ T cell responses to caspase-cleaved antigens derived from effector T cells undergoing apoptosis, may contribute to multiple sclerosis (MS) immunopathology.

**Methods:**

The percentage of autoreactive CD8^+^ T effector cells specific for various apoptotic T cell-associated self-epitopes (apoptotic epitopes) were detected in the peripheral blood and cerebrospinal fluid (CSF) by both enzyme-linked immunospot and dextramers of class I molecules complexed with relevant apoptotic epitopes. Moreover, the capacity of dextramer^+^ CD8^+^ T cells to produce interferon (IFN)-γ and/or interleukin (IL)-17 in response to the relevant apoptotic epitopes was evaluated by the intracellular cytokine staining. Cross-presentation assay of apoptotic T cells by dendritic cells was also evaluated *ex vivo*.

**Results:**

We found that polyfunctional (IFN-γ and/or IL-17 producing) autoreactive CD8^+^ T cells specific for apoptotic epitopes were represented in MS patients with frequencies significantly higher than in healthy donors. These autoreactive CD8^+^ T cells with a strong potential to produce IFN-γ or IL-17 in response to the relevant apoptotic epitopes were significantly accumulated in the CSF from the same patients. In addition, the frequencies of these autoreactive CD8^+^ T cells correlated with the disease disability. Cross-presentation assay revealed that caspase-cleaved cellular proteins are required to activate apoptotic epitope-specific CD8^+^ T cells *ex vivo*.

**Conclusion:**

Taken together, these data indicate that apoptotic epitope-specific CD8^+^ T cells with strong inflammatory potential are recruited at the level of the inflammatory site, where they may be involved in MS immunopathology through the production of high levels of inflammatory cytokines.

## Introduction

Multiple sclerosis (MS) is a chronic inflammatory demyelinating disease representing a major cause of neurological disability in the Western world [1]. Genetics, environmental factors - in particular the Epstein-Barr virus (EBV), and autoimmune steps are believed to be involved in MS development [1-4].

T helper (Th)1 cells were initially thought to be the only effector (memory) T cells (T_EM_) [5,6] capable of mediating inflammatory demyelination in MS [7]; however, the discovery of Th17 cells has led to the idea that IL-17 produced by CD4^+^ or CD8^+^ T cells specific to myelin proteins (likely in response to IL-23 and granulocyte monocyte-colony stimulating factor, GM-CSF) plays a preponderant role in the autoimmune demyelinating disease found in humans and mice [8-15]. The administration of IFN-β [16,17], the oral drug fingolimod (FTY-720) [18], or hematopoietic stem cell transplantation [19] has been associated with a reduction in Th17 responses that parallels clinical improvement.

Dendritic cells (DCs) that are also present in brain lesions and in the cerebrospinal fluid (CSF), can take up antigens (particularly from apoptotic cells), and migrate to the deep cervical lymph nodes for the priming or cross-priming of naive CD4^+^ or CD8^+^ T cells, respectively [20-26]. Recently, similar cross-presentation capacity and phagocytic function have been shown in all human lymphoid organ-resident DCs (including BDCA1^+^, BDCA3^+^ and the plasmocytoid DCs), confuting the observation proposing that BDCA1^+^ DCs (shown to be the homologues of mouse CD8^+^ DCs) cross-present antigens more efficiently than other blood DC subsets [27]. Whether DCs found in brain lesions or CSF belong to one or more of the DC subsets described above, or to monocyte-derived inflammatory DCs (recently demonstrated in various inflammatory environments [27]) is an important issue to investigate. We have previously shown that myelin basic protein (MBP)-specific T cells produce IFN-γ in response to autologous DCs loaded with apoptotic oligodendrocytes *in vitro*[28]. This result indicated that myelin-specific antigens presented within the apoptotic glial cells may be involved in the first relevant MS pathogenetic step. However, other steps may need to be carried out to initiate or aggravate the autoimmune process in the disease.

In the present study, we consider the fate of the large number of apoptotic cells resulting from the rapid turnover of effector T cells undergoing apoptosis after performing their functions in inflammatory diseases, and the possible role of these apoptotic cells in MS. In previous studies, we demonstrated that the proteome of apoptotic T cells include prominent caspase-cleaved cellular proteins (namely, fragments of actin cytoplasmic 1 (ACTB) [Swissprot: P60709], heterogeneous nuclear ribonucleoprotein (ROK) [Swissprot: P61978], lamin B1 (LAM1) [Swissprot: P20700], non-muscle myosin heavy chain 9 (MYH9) [Swissprot: P35579], vimentin (VIME) [Swissprot: P08670], proteasome component C2 (PSA1) [Swissprot: P25786], rho GDP dissociation inhibitor 2 (GDIS) [Swissprot: P50395], and 60S acidic ribosomal protein P2 (RLA) [Swissprot: P05387]), and a high proportion of distinct epitopes in these fragments (apoptotic epitopes) can be cross-presented by DCs via the classical major histocompatibility complex (MHC) class I pathway to a wide repertoire of autoreactive CD8^+^ T cells [25,29]. This observation led us to demonstrate that caspases within apoptotic cells can cleave fragments from long-lived proteins [30] that are strictly anchored to cellular structures (for example, the cytoskeleton); these fragments are then efficiently phagocytosed, processed, and cross-presented by DCs [25]. Further reports have confirmed the role of caspase cleavage in the processing and presentation of epitopes that are derived from apoptotic cells in different models [31-33]. In addition, apoptotic cells derived from activated T cells retain the expression of CD40 ligand (L) and can condition CD40^+^ DCs to acquire high capacities to prime or cross-prime autoreactive T cells [25,29,34]. This mechanism is consistent with the evidence that the signals provided by CD40L^+^ apoptotic cells and not those provided by conventional apoptotic cells (that generally induce immune tolerance or resolution of inflammation) facilitate the emergence of autoreactive T cell responses to apoptotic self-antigens [35,36]. In chronic HIV or hepatitis C virus (HCV) infections, the proportion of resulting autoreactive CD8^+^ T cells correlates with the proportion of circulating apoptotic CD4^+^ T cells *in vivo* and with the disease progression [25,29]. Research has suggested that the emergence and the maintenance of these responses contribute to amplification of the immunopathology through their capacity to produce high levels of inflammatory cytokines [25,29,34].

The aims of the present study are to determine whether CD8^+^ T cells specific for apoptotic self-epitopes are prominent in MS patients, to verify whether they have a distinct effector phenotype, to distinguish which of them is associated with the disease severity, and to ascertain the mechanisms whereby these responses are induced and maintained.

## Methods

### Study populations

For the present study, 26 consecutive HLA-A2^+^ MS patients (median age 40 years, range 19 to 61 years), who had presented for a diagnostic evaluation or relapse of MS at two neurological institutions during a 1-year period, were recruited; 20 of the patients were female. They were examined in accordance with the ethical guidelines of the 1975 Declaration of Helsinki and with a priori approval by the Ethics Committee of the Italian National Institute of Health. Written informed consent was obtained from all patients. The clinical and paraclinical characteristics of the patients included in this study are shown in Table 1. Inclusion criteria were as follows: MS diagnosis defined according to the McDonald criteria [37], the absence of an immunosuppressive therapy, and HLA-A2 positivity. All patients consented to the study and no patients were lost to follow up. The Expanded Disability Status Scale (EDSS) scores ranged from 1.0 to 6.0 (mean 2.6). The clinical course was classified as relapsing-remitting in twenty-four patients, whereas two patients had secondary-progressive MS. Ten patients were treated with glatiramer acetate or IFN-β, whereas sixteen patients did not receive any immunomodulating, immunosuppressive, or steroid therapy. Magnetic resonance imaging (MRI) was performed for each patient within 30 days from sampling. Nine patients presented with gadolinium-enhanced MRI lesions suggestive of blood-brain barrier damage. A lumbar puncture was performed in 15 of the 26 patients. With the exception of one subject, all displayed CSF oligoclonal immunoglobulin G (IgG) bands after CSF IgG isoelectric focusing in accordance with the recommended procedures. No patient was undergoing therapy with steroids or immunosuppressive drugs in the three months prior to sampling. All patients were subjected to clinical/paraclinical follow up from the time of diagnosis. Buffy coats from HLA-A2^+^ 27 sex and age-matched healthy donors (HDs) were provided by the blood bank of Dipartimento di Immunoematologia e Medicina Trasfusionale (Sapienza Università di Roma).

**Table 1:** Main demographic, clinical and MRI characteristics of HLA-A2+ patients

**Patient**	**Gender**	**Age**	**Duration (years)**	**Predominant symptoms**	**MRI Gd enhancement**	**EDSS**
1	F	48	1	motor	-	6
2	F	20	2	brainstem, ON	+	2
3	F	51	4	brainstem	+	3
4	F	29	3	cerebellar, sensitive	+	3
5	F	37	1	brainstem	-	1
6	F	53	7	brainstem	-	0
7	F	40	1	sensitive	-	2
8	M	39	1	cerebellar	-	1
9	F	40	2	ON	-	1
10	F	38	1	ON, sensitive	-	2
11	M	49	1	ON	-	1
12	F	46	1	sensitive	-	1
13	M	24	1	sensitive, ON	-	1
14	M	49	20	motor, ataxia	-	4
15	F	43	1	sensitive	-	2
16	F	34	2	sphincteric, ON	+	3
17	F	61	5	brainstem, vertigo	-	1
18	F	40	3	sensitive	-	3
19	M	43	2	motor	-	5
20	F	35	1	motor	-	1
21	F	43	5	motor	-	4
22	M	32	6	motor	+	7
23	F	53	4	motor, ataxia	+	4
24	F	19	1	brainstem	+	1
25	F	43	9	sensitive, brainstem	+	2
26	F	53	4	sensitive, motor	+	3

All patients had relapsing remitting MS, except for patients 1 and 14 who had secondary-progressive MS.

Legend: ON, optic neuritis; EDSS, expanded disability status scale; MRI Gd enhancement, nuclear magnetic resonance gadolinium enhancement.

### Synthetic peptides and reagents

Ninety-one HLA-2 binding peptides (nonamers or decamers) were derived from caspase-cleaved fragments of ACTB, ROK, LAM1, MYH9, VIME, PSA1, GDIS, and RLA as previously described (Additional file 1: Table S1). Seventeen 21-mer overlapping peptides spanning the entire human MBP sequence [Swissprot: P02686-5] were synthesized by high performance liquid chromatography (HPLC). The purity of peptides was determined by reverse-phase HPLC (Additional file 2: Table S2).

### Cell preparations

Peripheral blood mononuclear cells (PBMCs) were isolated and T cell lines were generated as previously described [23]. CD8^+^ T cells were purified from PBMCs by positive selection coupled to magnetic beads (Miltenyi Biotec, Bologna, Italy) [38]. Flow cytometry analysis demonstrated > 99% CD8^+^ cells in the positively purified population and < 5% in the CD8-depleted population. Spontaneous apoptosis of T cells from patients was determined by staining fresh PBMCs with fluorescein isothiocyanate (FITC)-labeled Annexin-V (Biolegend), propidium iodide (PI) (Biolegend, London, UK) and allophycocyanin (APC)-labeled anti-CD3 monoclonal antibody (mAb) (Biolegend). Immature (i)DCs were derived from peripheral monocytes and generated as described. Monocyte-derived iDCs were purified by positive selection with anti-CD14 mAb coupled to magnetic beads (MiltenyiBiotec). CD14^+^ cells were incubated for 5 days in Roswell Park Memorial Institute (RPMI) 1640 medium containing 10% FCS, 2 mM glutamine, 1% nonessential amino acids, 1% sodium pyruvate, 50 μg/ml kanamycin (Gibco BRL), 50 ng/mL recombinant (r)GM-CFS (PeproTech, DBA, Segrate - Milano, Italy), and 1000 U/mL rIL-4 (generously provided by A Lanzavecchia, Bellinzona, CH). Mature DCs were obtained by a 40-h stimulation of iDCs with soluble rCD40L molecules (Enzo Life Sciences, Vinci-Biotech, Vinci - Firenze, Italy). The definition of monocyte-derived DCs was based on their surface phenotype profile by staining with anti-CD14, anti-CD86 (Caltag Laboratories), anti-CD1a, anti-CD1c, anti-CD11c, anti-CD32, anti-CD80 (BD PharMingen, Milano, Italy) mAbs.

### Enzyme-linked immunospot assay

After stimulation with 12 independent pools of apoptotic peptides (Additional file 1: Table S1) or 16 single MBP peptides (Additional file 2: Table S2) PBMCs were tested by enzyme-linked immunospot (ELISPOT) assay. Briefly, 96-well millimeter high-affinity plates (Millipore Corporation, Bedford, MA, USA) were coated with 10 μg/mL of capture mAb against IFN-γ (BD PharMingen) at 4°C overnight. Plates were blocked for 2 h with blocking solution (PBS containing 2% BSA). A total of 1 × 10^5^ PBMCs were added to each well and stimulated for 18 h with peptides. Biotinylated anti-IFN-γ (BD PharMingen) diluted to 5 μg/mL in Blocking Solution was added and incubated for 2 h in 5% CO_2_ at 37°C. Plates were washed, incubated with alkaline phosphatase (AKP)-streptavidin (BD PharMingen) and developed with Sigmafast BCIP®/NBT (Sigma). The reaction was stopped by rinsing the plates with distilled water. Each well was then examined for positive dots. The number of dots in each well was counted by an ELISPOT reader system (AELVIS reader system). IFN-γ-secreting cells were expressed as IFN-γ spots per 1 × 10^6^ cells. The IFN-γ spot values were subtracted from the background, which was below 20 IFN-γ spots in 1 × 10^6^ cells for each test.

### Monoclonal antibody and dextramer staining

PBMCs were incubated with APC-labeled–HLA-A*0201 dextramer complexed to MYH9_478-486_ (QLFNHTMFI), MYH9_741-749_ (VLMIKALEL), VIME_78-87_ (LLQDSVDFSL), VIME_225-233_ (SLQEEIAFL) or ACTB_266-274_ (FLGMESCGI) peptides (Immudex, Copenaghen, Denmark). The incubation was performed in fluorescence-activated cell sorter (FACS) buffer (PBS containing 2% human AB serum) at room temperature for 10 minutes, followed by washing and further surface staining with FITC-labeled mAb to CD8 (eBioscience, San Diego, CA, USA), phycoerythrin-cyanine (PeCy)7-labeled mAb to PD-1 (eBioscience), AlexaFluor700-labeled mAb to CD69, PECF594-labeled mAb to HLA-DR, and a cocktail of labeled mAbs and reagents (APC-Cy7-labeled mAbs to CD4, CD14, CD16, CD19, and CD56 (Biolegend)) and Fixable Viability Dye eFluor 780 (eBioscience)) (dump channel), for 20 minutes at 4°C. Dextramer^+^ cells were analyzed within a CD8^+^ cell gate, whereas CD69^+^, HLA-DR^+^, or PD-1^+^ cells within dextramer^+^CD8^+^ cells, after exclusion of B cells, monocytes, natural killer T (NKT) cells, NK cells, CD4^+^ T cells (dump channel). Cells were acquired with LSRFortessa cytometer (Becton Dickinson) and analyzed with FlowJo software version 7.5.5 (Tree star, Inc. San Carlos, CA, USA).

### Intracellular cytokine staining

Cytokine production was analyzed by intracellular staining (ICS) assay. PBMCs were incubated with or without the relevant peptides (20 μg/mL) plus anti-CD28 mAb (4 μg/mL) (BD Biosciences) and Protein Transport Inhibitor Cocktail (500×) (Brefeldin A and Monensin) (eBioscience), or with Cell Stimulation Cocktail (500×) as positive control (PMA, ionomycin, brefeldin A and monensin) (eBioscience), for 18 h at 37°C. Cells were washed, and stained with APC-labeled-HLA-A*0201 dextramers complexed to corresponding peptides, PeCy7-labeled mAb to CD8 (Biolegend) and the dump channel reagents. Cells were fixed and permeabilized using Cytofix/Cytoperm solution (BD Biosciences) at 4°C for 20 minutes, re-washed with Perm Wash Buffer (BD Biosciences), and stained with different combinations of AlexaFluor700-labeled IL17A (Biolegend) and FITC-labeled anti-IFN-γ (Biolegend) for 20 minutes at 4°C. Cells were washed, acquired with LSRFortessa cytometer (Becton Dickinson, Milano, Italy) and analyzed with FlowJo software. IL-17, IFN-γ, or IL-17/IFN-γ-producing cells were analyzed in CD8^+^dextramer^+^ cells after exclusion of B cells, monocytes, NKT cells, NK cells, and CD4^+^ T cells (dump channel).

### Cross-presentation of apoptotic cells

Cloned CD8^+^CD95^+^ T cells (10 to 100 × 10^6^) were incubated in the presence or absence of 14 μg/mL caspase 3-inhibitor (C3I) (Z-DEVD-FMK), or a negative caspase control (Ctr) (K, Z-FA-FMK) (BD Pharmingen) for 1 h at 37°C in a 24-well plate. Then cells were induced to apoptosis by incubation with 500 ng/mL anti-Fas (anti-CD95 mAb (clone CH11), Upstate Biotechnology) for at least 6 h. Apoptotic cells were determined as described above. Finally, apoptotic T cells were isolated by positive selection with annexin-V coupled to magnetic beads (Miltenyi Biotec) as previously described. PBMCs were double-stained with dextramers and mAb to CD8 and cultured with iDCs that had been pulsed or not with apoptotic cloned T cells. After 6 to 8 h, cells were tested for their capacity to produce IL-17 and IFN-γ by ICS as described [25,29].

### Statistical analyses

The collected data were statistically analyzed using GraphPad Prism version 4 software (GraphPad Software). Comparison of the results for healthy donors (HDs) and patients was analyzed with the Mann-Whitney test. Dextramer^+^ CD8^+^ T cell frequencies in CSF and PBMCs were compared with the Wilcoxon matched pairs signed rank test. Linear regression analysis was performed to examine the correlation between tests and clinical data. The differences were considered significant at *P* < 0.05.

## Results

### Multispecific CD8^+^ T_EM_ cell responses to apoptotic epitopes

**Figure 1:**
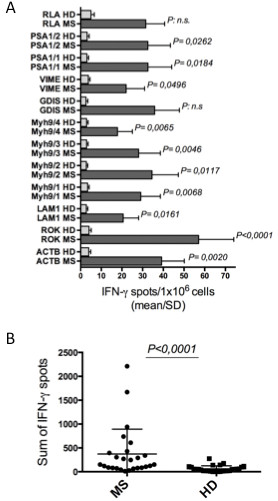
**CD8**^**+ **^**T cell multispecificity to apoptotic epitopes in healthy donors and multiple sclerosis patients. (A)** Mean number of IFN-γ spots formed by fresh CD8^+^ T effector memory (T_EM_) cells (by enzyme-linked immunospot assay) in response to 12 pools (see Additional file 1: Table S1A-C) of apoptotic self-epitopes in 26 HLA-A2^+^ patients with multiple sclerosis (MS) (dark bars) or 27 HLA-A2^+^ healthy donors (HDs) (clear bars). n.s., not significant. **(B)** Sum of IFN-γ spots formed by fresh CD8^+^ T_EM_ cells in response to all pools (see Additional file 1: Table S1A-C) of apoptotic self-epitopes in the single patients or HDs. Each circle represents a single patient or a single HD. Statistical analysis was performed with the Mann-Whitney test. RLA, 60S acidic ribosomal protein P2; PSA, proteosome component C3; VIME, vimentin; GDIS, GDP dissociation inhibitor 2; Myh9, non-muscle myosin heavy chain 9; LAM1, lamin B1; ROK, heterogeneous nuclear ribonucleoprotein; ACTB, actin cytoplasmic 1.

Freshly isolated CD8^+^ T cells from 26 consecutive HLA-A2^+^ patients with MS (Table 1) and 27 HDs were tested for the capacity to form IFN-γ spots in an ELISPOT assay within 4 to 6 h of contact either with 12 pools containing a total of 90 synthetic HLA-A2-binding apoptotic peptides (Additional file 1: Table S1) [25,29,35], or with overlapping peptides spanning the entire sequence of the MBP (Additional file 1: Table S2) [28]. Therefore, we defined these CD8^+^ cells as T_EM_, on the basis of their capacity to perform their effector functions promptly within a few hours of antigenic stimulus. Each peptide was tested in triplicate. The synthetic apoptotic peptides used were prepared according to the sequence of caspase-cleaved proteins that had been previously identified by the proteomic analyses of apoptotic T cells (for example, fragments of ACTB, ROK, LAM1, MYH9, GDIS, VIME, PSA1, and RLA) [25,29,35]. We found that the responses to apoptotic epitope by IFN-γ^+^CD8^+^ T_EM_ cells were significantly higher and wider in patients than in HDs (Figure 1). In particular, both the median number of IFN-γ spots formed by CD8^+^ T_EM_ cells from all MS patients or HDs in response to the single peptide pool (responsiveness) (Figure 1A), and the sum of IFN-γ spots formed in response to the total peptide repertoire by a single patient or HD (magnitude) (Figure 1B) were significantly higher in MS patients than in HDs. The HLA-restriction of these responses was demonstrated both by blocking responses with an appropriate anti-class I mAb and by determining that no response was observed in HLA-A2^–^ patients (data not shown). Notably, the responses to MBP epitopes by IFN-γ^+^ T_EM_ cells were not significantly different between patients and HDs (Additional file 3: Figure S1). No correlation was found between the ELISPOT responses either to apoptotic epitopes, or to MBP epitopes and EDSS, MRI grading, or clinical course of therapy (data not shown).

### Accumulation of apoptotic epitope-specific CD8^+^ T cells in CSF correlates with MS disability

**Figure 2:**
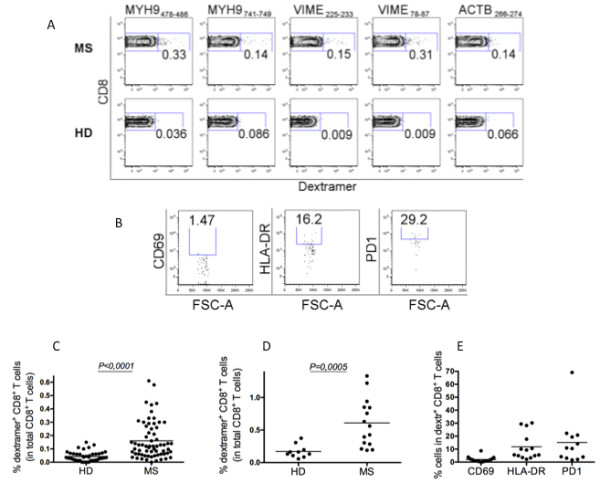
**Detection of peripheral CD8**^**+ **^**T cells specific to apoptotic epitopes in healthy donors and multiple sclerosis patients directly *in vivo*. (A)** Representative flow cytometry analysis of peripheral blood mononuclear cells, from a multiple sclerosis (MS) patient and a healthy donor (HD), were stained with a phycoerythrin-cyanine (PeCy7)-labeled mAb to CD8, allophycocyanin (APC)-labeled-HLA-A*0201 dextramers expressing the indicated apoptotic peptides, and the dump channel APC-Cy7-labeled reagents so as to exclude CD4^+^ T cells, monocytes, B cells, natural killer (NK) cells and dead cells from the analysis. Contour plot analyses show the percentage of CD8^+^dextramer^+^ cells. The percentage of cells is indicated in the appropriate quadrant. **(B)** Representative flow cytometry analysis of PBMCs stained with a pool of APC-labeled–HLA-A*0201 dextramer expressing relevant peptides, fluorescein isothiocyanate-labeled mAb to CD8, PeCy7-labeled mAb to PD-1, AlexaFluor700-labeled mAb to CD69, PECF594-labeled mAb to HLA-DR, and dump channel labeled reagents. Dot plot analyses show percentages of CD69^+^, HLA-DR^+^, or PD-1^+^ cells in gated CD8^+^dextramer^+^ cells. The percentage of cells is reported in each quadrant. **(C)** Percentage of CD8^+^dextramer^+^ cells specific to a single epitope in 10 HLA-A2^+^ HD and 15 HLA-A2^+^ patients (each symbol represents the percentage of CD8^+^dextramer^+^ cells specific to a single epitope in HDs or patients). **(D)** Sum of the percentages of all CD8^+^dextramer^+^ T cells detected in the single patient or HD (each symbol represents the sum of percentages of the five dextramers tested in the single individual). **(E)** Percentages of CD69^+^, HLA-DR^+^, or PD-1^+^ cells in CD8^+^dextramer^+^ T cells from MS patients. Statistical analysis was performed with the Mann-Whitney test. MYH9, non-muscle myosin heavy chain 9; VIME, vimentin; ACTB, actin cytoplasmic 1.

**Figure 3:**
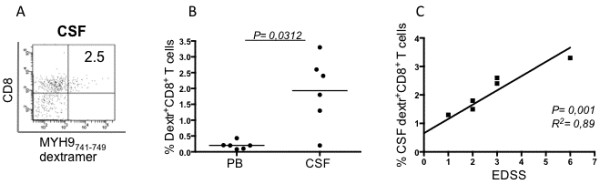
**Apoptotic epitope-specific CD8**^**+ **^**T cells are accumulated in cerebrospinal fluid and correlate with disease disability. (A)** Representative flow cytometry analyses of cerebrospinal fluid (CSF)-derived mononuclear cells from a multiple sclerosis (MS) patient. Cells were double-stained with a mAb to CD8 and dextramers expressing the indicated non-muscle myosin heavy chain 9 (MYH9) peptides. Dot plot analyses show the percentage of CD8^+^pentamer^+^ cells. The percentage of cells is indicated in the appropriate quadrant. **(B)** Percentages of peripheral blood (PB)- or CSF-derived CD8^+^dextramer^+^ cells isolated from seven HLA-A2^+^ MS patients. Statistical analysis was performed with the Wilcoxon matched pairs signed rank test. **(C)** Correlation between percentages of CSF-derived CD8^+^dextramer^+^ cells and disability, calculated on the Expanded Disability Status Scale (EDSS) (linear regression analysis).

First, we enumerated apoptotic epitope-specific CD8^+^ T cells in the peripheral blood of 15 HLA-A2^+^ MS patients (providing adequate numbers of PBMCs to extend the analyses to dextramer staining), as compared with 10 HDs, by using dextramers of HLA-A*0201 molecules complexed to apoptotic (ACTB_266-274_, MYH9_478-486_, MYH9_741-749_, VIME_78-87_, or VIME_225-233_) peptides (Figure 2). Control dextramers complexed to a non-natural irrelevant peptide were unable to stain CD8^+^ T cells in all samples tested (data not shown). All analyzed patients presented significantly higher frequencies of peripheral dextramer^+^CD8^+^ T cells than HDs, in terms of both responsiveness and magnitude (Figure 2A,C,D). In addition, a notable proportion of these apoptotic epitope-specific CD8^+^ T cells expressed late activation markers, namely HLA-DR and PD-1, suggesting that they are experienced T cells (Figure 2B,E). The percentage of apoptotic T cells circulating in PBMCs tended to be more elevated in patients than in HDs, though in a non-significant manner, thus, tempting us to suggest the possible relationship between apoptotic T cells and the emergence of CD8^+^ T cells specific to apoptotic epitopes (Additional file 4: Figure S2A,B) [25,29,35]: additional analyses in a larger cohort of patients are required to confirm this possibility. However, no correlations were observed between the frequency of peripheral dextramer^+^CD8^+^ T cells or expression of activation markers, and EDSS or MRI grading of MS patients tested (data not shown). Similar analyses were performed at the level of CSF-derived mononuclear cells obtained from seven MS patients. Because of the tiny number of cells isolated from the CSF, we chose to detect only MYH9_741-749_-specific CD8^+^ T cells by dextramers, on the basis of the observation that they were frequent in the periphery. Notably, among the MS patients tested, the frequency of MYH9_741-749_-specific CD8^+^ T cells was significantly higher in the CSF than in the periphery (Figure 3A,B). Importantly, the frequencies of CSF-derived CD8^+^ T cells specific to the apoptotic MYH9_741-749_ epitope were directly correlated with the disease disability (Figure 3C), suggesting that these cells are recruited in the inflammatory site and that they participate in the central CNS immunopathology.

### Polyfunctional (IFN-γ and IL-17) response by apoptotic epitope-specific CD8^+^ T cells

**Figure 4:**
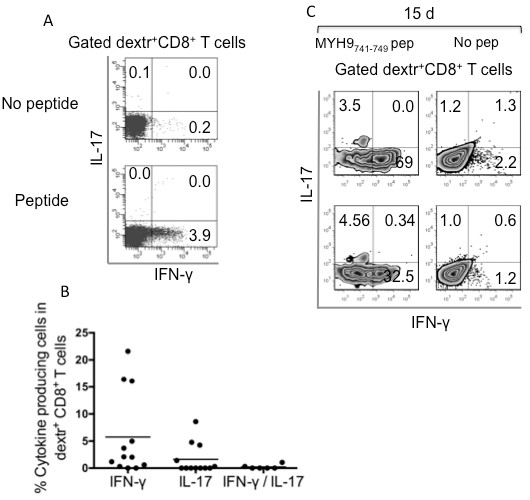
**Polyfunctional CD8**^**+ **^**T effector memory cells specific to apoptotic epitopes in multiple sclerosis patients. (A)** Representative flow cytometry analysis of peripheral blood mononuclear cells from a multiple sclerosis (MS) patient. Cells were incubated with or without the relevant peptides plus anti-CD28 mAb for 18 h at 37°C. Then cells were stained with dextramers complexed to corresponding peptides, phycoerythrin-cyanine-labeled mAb to CD8 and the dump channel reagents and processed for the detection of IL-17 and IFN-γ, by intracellular staining (ICS) assay with the relevant mAbs. Dot plot analyses are gated on CD8^+^dextramer^+^ cells and show percentages of cytokine-producing cells. The percentage of cells is reported in each quadrant. **(B)** Percentages of CD8^+^dextramer^+^ cells specific to the corresponding peptides producing IL-17, IFN-γ, or both within 18 h of contact with the relevant peptides in the 12 patients analyzed. **(C)** Representative flow cytometry analysis of an antigen-specific T cell line obtained upon 15 days stimulation with the non-muscle myosin heavy chain 9 (MYH9)_478-486_ epitope and IL-2. Cells were stained with mAb to CD8, the indicated dextramers, and were then stimulated or not with the relevant soluble peptide plus anti-CD28 mAb for detecting IL-17 and IFN-γ production by ICS assay, as described above. Contourplot analyses are gated on CD8^+^dextramer^+^ cells and show percentages of cytokine-producing cells. Similar results were obtained in six patients tested with different apoptotic epitopes.

**Figure 5:**
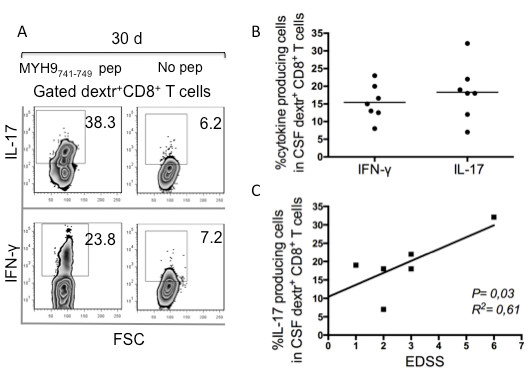
**Cerebrospinal fluid-derived apoptotic epitope-specific CD8**^**+ **^**T cells producing IL-17 are correlated with disability in multiple sclerosis. (A)** Representative flow cytometry analysis of an antigen-specific T cell line obtained upon 30 days of stimulation of cerebrospinal fluid (CSF)-derived mononuclear cells with the non-muscle myosin heavy chain 9 (MYH9)_741-749_ epitope and IL-2. Cells were stained with mAb to CD8, the indicated dextramers, and were then stimulated or not with the relevant soluble peptide plus anti-CD28 mAb for detecting IL-17 and IFN-γ production by intracellular staining (ICS) assay. Contour plot analyses are gated on CD8^+^dextramer^+^ cells and show percentages of cytokine-producing cells. **(B)** Percentage of CSF-derived MYH9_741-749_-specific T cell lines obtained from seven multiple sclerosis (MS) patients, as indicated in **(A)** producing IL-17 and IFN-γ in response to the relevant apoptotic peptide. CSF-derived T cell lines were stained with mAb to CD8, the indicated dextramers, and stimulated or not with the relevant soluble peptide plus anti-CD28 mAb for detecting IL-17 and IFN-γ production by ICS assay. **(C)** Correlation between percentage of CSF-derived MYH9_741-749_-specific T cell lines producing IL-17 and disability, as determined by the Expanded Disability Status Scale (EDSS) (linear regression analysis).

To determine whether subsets with different effector functions were present within the total CD8^+^dextramer^+^ T cells, we analyzed the frequencies of freshly isolated CD8^+^dextramer^+^ T cells that produced IFN-γ and/or IL-17 within a few hours of contact with the relevant peptides and optimal concentrations of anti-CD28 mAb, which served as a surrogate co-stimulatory signal. Undetectable cytokine production was observed when apoptotic epitope-specific CD8^+^dextramer^+^ T cells of 10 HLA-A2^+^ HDs were stimulated with this procedure (data not shown). By contrast, all apoptotic epitope-specific CD8^+^dextramer^+^ cells (derived from the peripheral blood of 12 MS patients) produced the cytokines tested in response to the relevant epitopes (polyfunctional responses) (Figure 4A,B) [6]. Peripheral dextramer^+^CD8^+^ T_EM_ cells particularly produced only IFN-γ (type-1 cells) or IL-17 (type-17 cells), whereas a minority of them simultaneously produced the two cytokines (type-1/17 cells) in response to relevant single epitopes (Figure 4B). To validate the antigen specificity of these peripheral dextramer^+^ cells, PBMCs from six randomly selected patients were cultured in the presence of the relevant peptide for 15 days in IL-2-conditioned medium *in vitro*, then stained with the relevant dextramer and anti-CD8, and tested for their capacity to respond to autologous PBMCs plus peptide. Under these conditions, the apoptotic antigen-specific CD8^+^ T cells continued to produce notable amounts of either IFN-γ or IL-17 in an antigen-specific manner, clearly confirming the antigen-specificity of these cells (Figure 4C). However, no correlation between the frequency of these peripheral cytokine-producing T_EM_ cells or the magnitude of the response by the cultured CD8^+^ T cells and any clinical parameter tested was observed (data not shown). To determine whether the lack of these correlations in the periphery was because of the recruitment of the majority of these cells at the level of the inflammatory site, we investigated the function of CSF-derived apoptotic epitope-specific CD8^+^ T cells. Because the tiny number of apoptotic epitope-specific CD8^+^dextramer^+^ T cells derived from the CSF did not allow prompt detection of cytokine production, we determined their functional potential upon two rounds of stimulation with autologous irradiated PBMCs in the presence of the relevant peptide for 30 days in IL-2-conditioned medium *in vitro*. Next, the cultured CSF cells were stained with the relevant dextramer and anti-CD8. They were tested for their capacity to respond to autologous PBMCs plus peptide. Under these conditions, we obtained a strong expansion of antigen-specific CD8^+^ T cells producing either IFN-γ or IL-17, suggesting that these cells are represented by CSF-derived memory cells selectively polarized towards a type-1 or type-17 profile (Figure 5A,B). Importantly, the CSF-derived apoptotic epitope-specific CD8^+^ T cells producing IL-17 were directly correlated with the EDSS (Figure 5C).

### Cross-presentation of apoptotic T cells *ex vivo*

**Figure 6:**
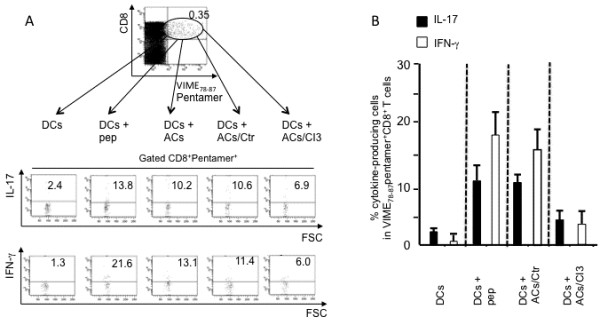
**Cross-presentation of naturally processed apoptotic epitopes to the related CD8**^**+ **^**T**_**EM **_**cells by dendritic cells. (A)** One representative of five experiments in which peripheral blood mononuclear cells from one MS patient were double-stained with mAb to CD8 and dextramers complexed with the indicated apoptotic epitope. These cells were then cultured with autologous dendritic cells (DCs) that had been pulsed or not with the relevant soluble peptide, apoptotic cloned T cells (ACs = apoptotic cells), ACs previously treated with a negative caspase control (Ctr = Z-FA-FMK), or ACs previously treated with the caspase 3 inhibitor (C3I = Z-DEVD-FMK). After 18 h, CD8^+^dextramer^+^ cells were tested for their capacity to produce the different cytokines indicated by the intracellular staining assay. Dot plots are gated on CD8^+^dextramer^+^ cells and show percentages of the different cytokine-producing cells in each quadrant. **(B)** Cumulative experiments in five independent patients, performed as described in **(A)**, showing the mean percentages of cells producing IL-17 (solid bars) and IFN-γ (open bars) in CD8^+^dextramer^+^ cells in response to the indicated stimuli: autologous DCs alone; DCs pulsed with the vimentin (VIME)_78-87_ peptide; DCs pulsed with ACs, which had been previously treated with a negative caspase control (Z-FA-FMK); or DCs pulsed with ACs, which had been previously treated with the C3I (Z-DEVD-FMK).

To verify if the cross-presentation mechanism plays a role in the activation of apoptotic epitope-specific CD8^+^ T cells, we evaluated the capacity of fresh dextramer^+^CD8^+^ T_EM_ cells to produce IFN-γ or IL-17 within a few h of contact with HLA-A2^+^ DCs that had been previously pulsed with apoptotic T cells. Under these conditions, dextramer^+^CD8^+^ T_EM_ cells promptly produced IFN-γ or IL-17 *ex vivo* (Figure 6A). The cross-presentation resulted in a marked decrease in IFN-γ or IL-17 production when apoptotic cells had been previously treated with the selective C3I, but not when treated with an appropriate negative Ctr (Figure 6A). This phenomenon was independently confirmed in five patients (Figure 6B). Although DCs are known to endogenously express high levels of the ubiquitous (long-lived) cellular proteins (ACTB, ROK, LAM1, MYH9, VIME, or PSA1) [25], DCs alone were unable to directly stimulate the related specific CD8^+^ T cells, thus supporting the concept that cross-presentation of these proteins requires caspase-dependent cleavage (Figure 6A,B).

## Discussion

Here we demonstrate for the first time that the multispecificity, magnitude, and polyfunctional (type-1 and type-17) strength of CD8^+^ T_EM_ cell responses directed to apoptotic self-epitopes were significantly higher in MS patients as compared with HDs. Importantly, the frequencies of the CSF-derived apoptotic epitope-specific CD8^+^dextramer^+^ T cells were significantly higher than those of the peripheral counterparts, and correlated with the disease disability. These CSF-derived apoptotic antigen-specific CD8^+^ T cells significantly expanded and produced high levels of pro-inflammatory cytokines (IFN-γ and IL-17) in response to antigenic stimulus *in vitro*, and those with the potential to produce IL-17 in response to the relevant apoptotic epitope were correlated with the disease disability. By contrast, the peripheral apoptotic antigen-specific dextramer^+^CD8^+^ T_EM_ cells that produced IFN-γ or IL-17 in response to the apoptotic epitopes analysed *ex vivo*, as well as upon 15 d of antigen-stimulation *in vitro*, did not correlate with any clinical parameter. Altogether these data suggest that apoptotic self-antigen-specific T cells with strong inflammatory potential are recruited at the level of the inflammatory site, where they largely expand under certain inflammatory conditions and may contribute, through the production of high levels of inflammatory cytokines, to immunopathology and chronic evolution of MS. In addition, these results emphasize the concept that the CSF-confined immune cell responses reflect the immunologic setting at the level of an inflammatory process occurring in the CNS, to a greater extent than those in the periphery.

Notably, our finding of no correlation between MBP-specific responses and MS does not lend support to defining MBP as a major immunological target in MS. However, it is unlikely that the T cell responses to apoptotic antigens represent the event initiating the CNS pathology. Indeed, our previous studies demonstrated that these responses require stimulation by a considerable number of apoptotic cells that derive from pre-existing activated T cells, such as the virus-specific T cells that are generated during HIV or HCV infections [25,29]. In the case of MS, the primary activated T cells may be specific for (but as of yet unknown) CNS-derived self-antigens or even pathogens (correlated with MS) that start the immunological cascade leading to MS, and consequently may induce apoptotic antigen-specific T cells by providing them with the first boost of apoptotic antigens. According to this scenario, the apoptotic antigen-specific T cell responses would represent an epiphenomenon of the responses that have initiated the inflammatory program. They may strongly contribute to amplify and sustain CNS damage through a vicious cycle, providing continuous waves of apoptotic antigens upon performing their pro-inflammatory activity, as indicated by both the substantial accumulation of these cells with strong inflammatory potential in CSF and their correlation with the disease disability. The finding that the same responses are operative in various chronic infections (HIV and HCV [25,29]), and in different autoimmune diseases, such as rheumatoid arthritis (manuscripts in preparation), suggests that they may support a general mechanism in several immunopathological conditions. It will be of interest to verify whether a genetic background predisposing to autoimmunity harbors variants that may foster this mechanism.

The observation that caspase-cleavage of apoptotic antigens is required to activate the related CD8^+^ T_EM_ cells by cross-presentation *ex vivo* indicates *t*hat these autoreactive CD8^+^ T cells may contribute to the CNS damage through the production of pro-inflammatory IFN-γ and IL-17 cytokines upon cross-presentation of the huge number of CD40L^+^ apoptotic cells (infiltrating inflamed tissues [39]), rather than by the direct killing of CNS cells [10], [25,34,36]. We cannot exclude the possibility that apoptotic CNS cells (for example, oligodendrocytes) may also amplify this phenomenon in an inflammatory context, because they might potentially generate similar caspase-cleaved antigenic fragments. In addition, other mechanisms may contribute to establishing the chronic immunopathological processes. Recently, the increased frequency of EBV-specific CD8^+^ T cells interacting with EBV-infected plasma cells in white matter has been associated with the active phase of MS [40]. Moreover, several independent memory T cells (for example, those that are specific to a multitude of pathogens normally circulating in the lymphoid tissues), which are stimulated in a bystander fashion, can be recruited in an inflammatory site where they can perform effector functions [41-43].

Finally, our results may have clinical implications. Further studies are needed to ascertain whether polyfunctional CD8^+^ T cells that are specific to apoptotic epitopes could predict relapses in MS or worsening of the disease. Accordingly, appropriate therapies could be administered earlier and, in so doing, limit the disease severity in patients who are expected to experience the clinical evolution of MS and neurological disability.

## Conclusions

The demonstration that mixed polyfunctional (type-1 and -17) CD8^+^ T_EM_ cells specific for apoptotic T cell-associated self-epitopes are recruited in the CSF of MS patients and are associated with the clinical score of disease disability, suggests that apoptotic self-antigen-specific T cells with strong inflammatory potential largely expand at the level of the inflammatory site, and may contribute, through the production of high levels of inflammatory cytokines, to MS immunopathology. Cross-presentation of caspase-cleavage of apoptotic antigens is required to activate these autoreactive CD8^+^ T_EM_ cells *ex vivo*, suggesting *t*hat the latter may participate to the CNS damage through the production of pro-inflammatory IFN-γ and IL-17 cytokines upon cross-presentation of the huge number of apoptotic cells present in the inflamed tissue.

## Abbreviations

ACTB: Actin cytoplasmic 1; APC: Allophycocyanin; BSA: Bovine serum albumin; C3I: Caspase 3-inhibitor; CNS: Central nervous system; CSF: Cerebrospinal fluid; Ctr: Caspase control; DC: Dendritic cell; EBV: Epstein-Barr virus; EDSS: Expanded Disability Status Scale; ELISPOT: Enzyme-linked immunospot; FACS: Fluorescence-activated cell sorting; FCS: Fetal calf serum; FITC: Fluorescein isothiocyanate; GDIS: GDP dissociation inhibitor 2; GM-CSF: Granulocyte monocyte-colony stimulating factor; HCV: Hepatitis C virus; HD: Healthy donor; HPLC: High performance liquid chromatography; ICS: Intracellular staining; iDC: Immature dendritic cell; IFN: Interferon; IgG: Immunoglobulin G; IL: Interleukin; L: Ligand; LAM1: Lamin B1; mAb: Monoclonal antibody; MBP: Myelin basic protein; MHC: Major histocompatibility complex; MRI: Magnetic resonance imaging; MS: Multiple sclerosis; MYH9: Non-muscle myosin heavy chain 9; NKT: Natural killer T; PBMC: Peripheral blood mononuclear cell; PBS: Phosphate-buffered saline; PeCy: Phycoerythrin-cyanine; PI: Propidium iodide; PSA1: Proteasome component C2; RLA: 60S acidic ribosomal protein P2; ROK: Heterogeneous nuclear ribonucleoprotein; RPMI: Roswell Park Memorial Institute; TEM: T effector memory; Th: T helper; VIME: Vimentin.

## Competing interests

The authors declare that they have no competing interests.

## Authors’ contributions

FL participated to the conception of the study and recruited patients and samples. EP, MPA, RM, and VA recruited patients and samples. HM, AC, and DF performed immunology experiments (flow cytometry and ICS analyses, ELISPOT analyses, T cell sorting and cloning, cross-presentation….). JS, and AS designed and synthesized peptides. MS participated to the conception of the study and recruited patients and samples. VB conceived the study and wrote the manuscript. All authors read and approved the final manuscript.

## Supplementary Material

Additional file 1: Table S1 A-C HLA-A2 binding peptides derived from apoptotic cell-associated proteins. Click here for file

Additional file 2: Table S2 HLA-A2 binding peptides derived from protein. Click here for file

Additional file 3: Figure S1 CD8^+^ T cell response to MBP in HD and MS patients. Mean number of IFN-γ spots by fresh CD8^+^ T effector memory (T_EM_) cells (by enzyme-linked immunospot (ELISPOT) assay) formed in response to the single overlapping myelin basic protein (MBP) peptides (see Additional file 2: Table S2) in 27 healthy donors (HD) (open bars) and 26 multiple sclerosis (MS) patients (solid bars). Statistical analysis, as performed using the Mann-Whitney test, showed no significant difference. Click here for file

Additional file 4: Figure S2 Increased number of circulating apoptotic T cells in multiple sclerosis (MS) patients. (**A**) Percentage of early apoptotic Annexin V ligand^+^ PI^-^ CD3^+^ T cells in healthy donors (HD) and MS patients. Statistical analysis was performed using the Mann-Whitney test. (**B**) Representative flow cytometry analysis of apoptotic Annexin V ligand (L)^+^ PI^-^ CD3^+^ T cells in an MS patient. Fresh peripheral blood mononuclear cells (PBMCs) were stained with Annexin-V, PI, and anti-CD3 mAb. Dot plot analyses are gated on CD3^+^ cells and show percentage of Annexin V (L)^+^ cells. Click here for file
