# Supplementary material for: Increased CD8+ T cell responses to apoptotic T cell-associated antigens in multiple sclerosis
Source: J Neuroinflammation. 2013 Jul 27;10:94. doi: 10.1186/1742-2094-10-94 (PMC3734107; doi:10.1186/1742-2094-10-94)
Supplement: Additional file 2: Table S2 — HLA-A2 binding peptides derived from protein. [file 1742-2094-10-94-S2.pdf]

Supplementary Table 2: HLA-A2 binding peptides derived from MBP protein				
**Peptide**	**Organism**	**1st pos**	**Sequence**	**Leght**
1	Human	1	ASQKRPSQRHGSKYLATAST	20
2	Human	13	KYLATASTMDHARHGFLPRH	20
3	Human	23	HARHGFLPRHRDTGILDSIG	20
4	Human	33	RTDGILDSIGRFFGGDRGAP	20
5	Human	43	RFFGGDRGAPKRGSGKDSHH	20
6	Human	55	GSGKDSHHPARTAHYGSLPQ	20
7	Human	65	RTAHYGSLPQKSHGRTQDEN	20
8	Human	74	QKSHGRTQDENPVVHFFKNI	20
9	Human	84	NPVVHFFKNIVTPRTPPPSQ	20
10	Human	94	VTPRTPPPSQGKGRGLSLSR	20
11	Human	104	GKGRGLSLSRFSWGAEGQPR	20
12	Human	114	FSWGAEGQPRPGFGYGGRASD	20
13	Human	124	GFGYGGRASDYKSAHKGFKG	20
14	Human	134	YKSAHKGFKGVDAQGTLSKI	20
15	Human	144	VDAQGTLSKIFKLGGRDSRS	20
16	Human	153	IFKLGGRDSRSGSPMARR	18
