# Supplementary figures and images for: Increased CD8+ T cell responses to apoptotic T cell-associated antigens in multiple sclerosis
Source: J Neuroinflammation. 2013 Jul 27;10:94. doi: 10.1186/1742-2094-10-94 (PMC3734107; doi:10.1186/1742-2094-10-94)

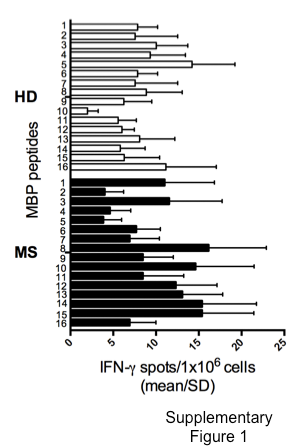

Supplement: Additional file 3: Figure S1 — CD8+ T cell response to MBP in HD and MS patients. Mean number of IFN-γ spots by fresh CD8+ T effector memory (TEM) cells (by enzyme-linked immunospot (ELISPOT) assay) formed in response to the single overlapping myelin basic protein (MBP) peptides (see Additional file 2: Table S2) in 27 healthy donors (HD) (open bars) and 26 multiple sclerosis (MS) patients (solid bars). Statistical analysis, as performed using the Mann-Whitney test, showed no significant difference. [file 1742-2094-10-94-S3.tiff]

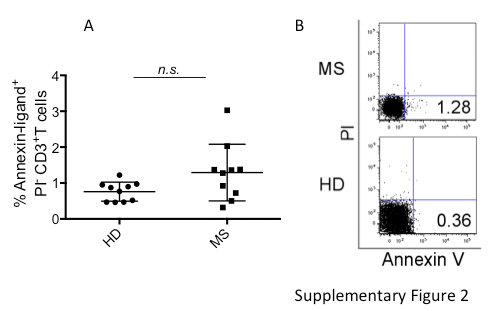

Supplement: Additional file 4: Figure S2 — Increased number of circulating apoptotic T cells in multiple sclerosis (MS) patients. (A) Percentage of early apoptotic Annexin V ligand+ PI- CD3+ T cells in healthy donors (HD) and MS patients. Statistical analysis was performed using the Mann-Whitney test. (B) Representative flow cytometry analysis of apoptotic Annexin V ligand (L)+ PI- CD3+ T cells in an MS patient. Fresh peripheral blood mononuclear cells (PBMCs) were stained with Annexin-V, PI, and anti-CD3 mAb. Dot plot analyses are gated on CD3+ cells and show percentage of Annexin V (L)+ cells. [file 1742-2094-10-94-S4.tiff]
